# Photography in the Great War: The Ethics of Emerging Medical Collections for the Great War

**DOI:** 10.5195/jmla.2024.1657

**Published:** 2024-01-16

**Authors:** Misti Thornton

**Affiliations:** 1 mthornton@umc.edu, University of Mississippi Medical Center, Jackson, MS

Photography in the Great War: The Ethics of Emerging Medical Collections for the Great War gives a brief history on photography and how it was used in partnership with surgeons and photographers to provide aid for disfigured servicemen who served in World War I. Focused mainly overseas during World War I, the book reflects how progress was made in the art of photography as technology changed, and how photography may have benefited the treatment of wounded soldiers.

The book is divided into five chapters. Chapter one describes the early history of facial injury photography. This chapter sets the groundwork for the proceeding chapters explaining how photography was used to document disfigurements and treatments.

The second chapter describes the technological advances such as electricity, along with the role photography played in documenting surgical cases of facially disfigured servicemen. The chapter also explains how photographs were published in scientific journal articles to illustrate treatments used during wartime for facial disfigurement surgeries that could be replicated throughout the world.

Alternate health care is the focus of chapter three. Photography was used in performing alternate health care therapies, especially in Italy where healthcare was different from other countries due to lack of funding. Nurses also played a role in photographing disfigured soldiers.

The psychological impact of the soldierâ€™s homecoming is detailed in chapter four. In this chapter, photography is used to influence and provide opportunities for the injured soldiers. This chapter discusses how men had to re-establish themselves in society, and how photography benefited their recovery as a means of therapy. Photography offered a reprieve for the soldiers coming home as a way of forgetting the trauma of war.

Medical and dental photographs of facial disfigurations are highlighted in the last chapter. The photographs that make up these collections tell of the social activity and provide an opportunity for surviving relatives to view them. This process was made easier with the digitization of some of these important wartime photographic collections.

Bate's monograph gives an excellent account of service men returning from war, wounded, disfigured and alienated. He cites family and public support determine how well veterans re-integrate into society. Bates draws attention to the ethical guidelines in using these photographs such as privacy and anonymity. He is also a strong advocate for preserving medical photographs, and proponent of protecting the private lives of the patients captured in these medical photographic collections. I would recommend Photograph in the Great War: The Ethics of Emerging Medical Collections for the Great War for those that are interested in the history of photography, and medical history.

